# Zika Virus Detection with 2013 Serosurvey, Mombasa, Kenya

**DOI:** 10.3201/eid2607.191363

**Published:** 2020-07

**Authors:** Elizabeth Hunsperger, Dennis Odhiambo, Albina Makio, Moshe Alando, Melvin Ochieng, Victor Omballa, Peninah Munyua, Godfrey Bigogo, M. Kariuki Njenga, Marc-Alain Widdowson

**Affiliations:** US Centers for Disease Control and Prevention, Nairobi, Kenya (E. Hunsperger, P. Munyua, M.-A. Widdowson);; Kenya Medical Research Institute, Nairobi, Kenya (D. Odhiambo, A. Makio, M. Alando, M. Ochieng, V. Omballa, G. Bigogo);; Washington State University, Pullman, Washington, USA (M.K. Njenga);; Institute of Tropical Medicine, Antwerp, Belgium (M.-A. Widdowson)

**Keywords:** Kenya, Zika, Zika virus, dengue virus, detection, viruses, vector-borne infections, serology, serosurvey, Mombasa, IgM

## Abstract

Acute Zika virus (ZIKV) infection has not been confirmed in Kenya. In 2018, we used specimens collected in a 2013 dengue serosurvey study in Mombasa to test for ZIKV IgM. We confirmed specific ZIKV IgM positivity in 5 persons. These results suggest recent ZIKV transmission in the coastal region of Kenya.

Zika virus (ZIKV), an arbovirus belonging to the family *Flaviviridae*, was initially isolated in 1947 from the serum of a pyrexic sentinel rhesus macaque in the canopy of the Zika Forest in Uganda (*1*). In 1970, ZIKV antibodies were reported in humans in Kenya, using immunoassays with limited sensitivity and specificity (*2*); since then, only past infections have been reported (*3*,*4*).

In 2015, the emergence of ZIKV in the Americas (*5*) raised the possibility that ZIKV infections were also occurring in Kenya but were undetected due to mild or asymptomatic infections and rudimentary birth defect surveillance. In 2016, Cape Verde and, in 2017, Angola reported ZIKV outbreaks associated with microcephaly. Genome sequencing from Angola identified the similarity of the Asian lineage of ZIKV to the American strain (*6*); this evidence implies that the African lineage may be less likely to cause microcephaly, thus explaining the few reported number of ZIKV-associated microcephaly cases in Africa (*7*).

To understand prior transmission of ZIKV in Kenya, in 2018, we used specimens collected in 2013 for a dengue serosurvey from Mombasa amidst a dengue outbreak. During May 3–11, 2013, serum specimens were collected from 1,500 consenting household members in 986 randomly selected households (*8*). Specimens were tested for dengue virus (DENV) by real-time reverse transcription PCR (rRT-PCR) and DENV IgM ELISA (InBios International, Inc., https://inbios.com). Concurrent with this serosurvey, hospital surveillance of suspected dengue patients was established (*8*). 

For our study, we used only the DENV-negative serum specimens and tested them for ZIKV by Centers for Disease Control and Prevention rRT-PCR and ZIKV IgM antibody capture ELISA (MAC ELISA). A ZIKV MAC ELISA specimen with a positive-to-negative ratio >3.00 was positive and a ratio of 2–2.99 was equivocal. We confirmed all positive specimens with 90% plaque reduction neutralization test (PRNT_90_) against ZIKV strain MR766 (African lineage) and DENV (ChimeraVax; Sanofi Pasteur, https://www.sanofi.com) (*9*). We defined recent ZIKV infection by a positive result in the ZIKV MAC ELISA and a PRNT_90_ titer of ZIKV that was 4-fold higher than the titer of DENV. The institutional review board at US Centers for Disease Control and Prevention and Kenya Medical Research Institute Scientific Ethics Review Unit approved the study.

We identified a total of 745 DENV-negative persons from the dengue serosurvey (n = 704) and hospital surveillance (n = 41); median age was 28 (range 0–94) years. None were positive by ZIKV rRT-PCR. Thirty-four (4.6%) were positive by ZIKV MAC ELISA, 7 captured in hospital surveillance; 24 (3.2%) were equivocal and 687 (92.2%) negative. Of the 34 ZIKV MAC ELISA positives, the ZIKV PRNT_90_ assay confirmed 5 (15.1%, 4 from serosurvey and 1 hospital) as ZIKV, 7 (21.2%) as DENV, 3 (9.1%) as cross-reactive to both viruses, and 18 (54.5%) as negative for ZIKV and DENV; 1 (2.9%) could not be confirmed due to insufficient sample (Table). Of the 5 ZIKV MAC ELISA–positive patients confirmed by ZIKV PRNT_90_, median age was 57 (range 50–70) years.

**Table:** Test results for 16 participants in a 2013 serosurvey in Kenya whose samples were positive for ZIKV by testing conducted in 2018*

Participant no.	Age, y	CDC ZIKV MAC ELISA P/N ratio (IgM)	ZIKV PRNT_90_ titer	DENV PRNT_90_ titer	Participant type	Final interpretation of case
2	63	3.56	1:20	<1:20	Serosurvey	ZIKV
3	50	10.00	>1:320	<1:20	Serosurvey	ZIKV
5	70	3.17	1:20	<1:20	Serosurvey	ZIKV
10	NA	3.51	1:20	<1:20	Hospital	ZIKV
31	51	5.50	>1:320	1:20	Serosurvey	ZIKV
4	45	3.26	<1:20	1:20	Serosurvey	DENV
7	36	8.65	1:20	1:320	Serosurvey	DENV
20	58	13.18	1:20	1:320	Serosurvey	DENV
25	45	3.70	1:20	>1:320	Serosurvey	DENV
26	27	5.00	1:80	>1:320	Serosurvey	DENV
27	42	3.20	1:80	1:320	Serosurvey	DENV
33	30	10.70	1:20	1:80	Serosurvey	DENV
1	32	4.49	1:20	1:20	Serosurvey	Cross-reactive
9	27	3.82	1:20	1:20	Serosurvey	Cross-reactive
30	58	3.20	>1:320	>1:320	Serosurvey	Cross-reactive
6	41	16.35	1:20	ND	Serosurvey	Inconclusive

*CDC, Centers for Disease Control and Prevention; DENV, dengue virus; MAC, IgM antibody capture; NA, not available; ND, not done due to insufficient specimen; PRNT_90_, plaque reduction neutralization test 90%; ZIKV, Zika virus.

We identified 5 persons sampled in 2013 who were positive for ZIKV IgM and confirmed by PRNT_90_. Of note, the positive participants (median age 57 years) were older than the sample group tested (median age 29 years). The level of transmission of ZIKV in Kenya is unknown, although Kenya has the competent vector (*Aedes aegypti* mosquitoes), and parts of Kenya are ecologically similar to the Zika forest (*1*). ZIKV has not been detected in Kenya despite recent intensive follow-up of pregnant women in coastal Kenya (*10*). Our study suggests that ZIKV may circulate in Mombasa and cause asymptomatic disease not captured in hospital surveillance systems. In addition, severely ill ZIKV patients might not have been identified from the original dengue study because it only captured clinical endpoints related to severe dengue.

We found 1 participant positive by ZIKV MAC ELISA but negative by ZIKV and DENV PRNT_90_, suggesting the presence of another co-circulating cross-reactive flavivirus in this region of Kenya (e.g., West Nile virus). This finding merits further investigation to determine all circulating flaviviruses in Mombasa. ZIKV transmission season in Kenya most likely coincides with other arboviruses that share the same vector. Of interest, titers of neutralizing antibodies against ZIKV were low for 3 of the 5 positive participants (1:20 PRNT_90_ titer), typically observed in acute infections (<10 days after onset of illness). Two participants had high neutralization titers (>1:320), suggesting recent infection (within the previous 90 days).

Our study had some limitations. It was conducted in 2018, so specimens had been archived for 5 years. We also had incomplete demographic and clinical data and could not discount concurrent DENV infections. Finally, we might have underestimated ZIKV positives because of weak neutralization by IgM.

In conclusion, ZIKV may have circulated at low levels in Kenya in 2013. More research is needed to evaluate current ZIKV circulation and characterize other co-circulating flaviviruses. Enhanced surveillance systems, including for microcephaly and other birth defects, could capture ZIKV patients and determine the epidemiology of ZIKV African lineage in this country.
